# THE IMPACT OF COMPANION ROBOTIC PETS ON WELL-BEING AMONG COMMUNITY-DWELLING WOMEN AGED 65 AND OLDER

**DOI:** 10.1093/geroni/igae098.2908

**Published:** 2024-12-31

**Authors:** Suk-Young Kang, Jeungkun Kim, Melanie Kuhl

**Affiliations:** Binghamton University, Binghamton, New York, United States; Kangnam University, Yongin-Si, Kyonggi-do, Republic of Korea; Binghamton University, Binghamton, New York, United States

## Abstract

This study investigates the impact of companion robotic pets on depression, anxiety, loneliness, and self-rated physical health in community-dwelling women aged 65 and older. Employing a time series design for baseline stability and treatment effects (Knapp, 2018, p.237), 120 respondents from the local Office for Aging were screened, and 45 with depressive symptoms (GDS-15) were selected. Selected participants received a dog or cat robotic pet during a home visit, with follow-up visits scheduled one month later, constituting three data collection points. Analyzing depression data, baseline mean was 9.16 (SD = 2.70). Pre-test mean increased slightly to 9.40 (SD = 2.75), but significantly decreased to 5.29 (SD = 3.18) post-test. For anxiety, baseline mean was 13.18 (SD = 5.27). Pre-test mean decreased to 12.24 (SD = 4.99), and significantly decreased to 7.33 (SD = 4.35) post-test. Loneliness revealed a baseline mean of 4.31 (SD = 1.58), a pre-test mean of 4.62 (SD = 1.43), and a significant decrease to 3.13 (SD = 2.04) post-test. All reductions were statistically significant. Conversely, self-rated physical health exhibited a baseline mean of 1.44 (SD =.81), a pre-test mean of 1.42 (SD =.84), and a significant increase to 1.73 (SD =.84) post-test. These results affirm that companion robotic pets may enhance mental well-being in older women dealing with depression and anxiety. Furthermore, companion robotic pets may alleviate feelings of loneliness and contribute to improved self-rated physical health, substantiating their overall beneficial impact.

